# A Study on the Outcome of Targeted Temperature Management Comparing Cardiac Arrest Patients Who Received Bystander Cardiopulmonary Resuscitation With Those Who Did Not, Using the Nationwide TIMECARD Multicenter Registry

**DOI:** 10.3389/fmed.2022.779781

**Published:** 2022-04-13

**Authors:** Fang-Yu Liou, Min-Shan Tsai, Li-Kuo Kuo, Hsin-Hui Hsu, Chih-Hung Lai, Kun-Chang Lin, Wei-Chun Huang

**Affiliations:** ^1^Department of Critical Care Medicine, Kaohsiung Veterans General Hospital, Kaohsiung, Taiwan; ^2^Education Center, National Cheng Kung University, Tainan, Taiwan; ^3^Department of Emergency Medicine, National Taiwan University Medical College and Hospital, Taipei, Taiwan; ^4^Department of Critical Care Medicine, MacKay Memorial Hospital, Taipei, Taiwan; ^5^Department of Medicine, Mackay Medical College, New Taipei City, Taiwan; ^6^Department of Critical Care Medicine, Changhua Christian Hospital, Changhua City, Taiwan; ^7^Cardiovascular Center, Taichung Veterans General Hospital, Taichung, Taiwan; ^8^Institute of Clinical Medicine, National Yang-Ming Chiao-Tung University, Taipei, Taiwan; ^9^Department of Physical Therapy, Fooyin University, Kaohsiung, Taiwan; ^10^School of Medicine, National Yang-Ming Chiao-Tung University, Taipei, Taiwan

**Keywords:** cardiac arrest, targeted temperature management, bystander cardiopulmonary resuscitation, witnessed collapse, electrical discharge, coronary intervention

## Abstract

**Background and Purpose:**

Targeted temperature management (TTM) is associated with decreased mortality and improved neurological function after cardiac arrest. Additionally, studies have shown that bystander cardiopulmonary resuscitation (BCPR) doubled the survival of patients with out-of-hospital cardiac arrest (OHCA) compared to patients who received no BPCR (no-BCPR). However, the outcome benefits of BCPR on patients who received TTM are not fully understood. Therefore, this study aimed to investigate the outcome differences between BCPR and no-BCPR in patients who received TTM after cardiac arrest.

**Methods:**

The Taiwan Network of Targeted Temperature Management for Cardiac Arrest (TIMECARD) multicenter registry established a study cohort and a database for patients receiving TTM between January 2013 and September 2019. A total of 580 patients were enrolled and divided into 376 and 204 patients in the BCPR and no-BCPR groups, respectively.

**Results:**

Compared to the no-BCPR group, the BCPR group had a better hospital discharge and survival rate (42.25 vs. 31.86%, *P* = 0.0305). The BCPR group also had a better neurological outcome at hospital discharge. It had a higher average GCS score (11.3 vs. 8.31, *P* < 0.0001) and a lower average Glasgow–Pittsburgh cerebral performance category (CPC) scale score (2.14 vs. 2.98, *P* < 0.0001). After undertaking a multiple logistic regression analysis, it was found that BCPR was a significant positive predictor for in-hospital survival (OR = 0.66, 95% CI: 0.45–0.97, *P* = 0.0363).

**Conclusions:**

This study demonstrated that BCPR had a positive survival and neurological impact on the return of spontaneous circulation (ROSC) in patients receiving TTM after cardiac arrest.

## Introduction

Post-cardiac arrest care plays a crucial role in the functional recovery of patients after cardiac arrest. Targeted temperature management (TTM) is an important post-cardiac arrest neuroprotective treatment for patients after the return of spontaneous circulation (ROSC) (1). Although the pharmacologic mechanisms are not fully understood, there is a possibility of attenuating post-arrest reperfusion injury to therapeutic hypothermia by reducing cerebral metabolism, thereby reducing the release of excitatory amino acids and the production of oxygen free radicals and restoring the mechanism of normal intracellular signaling (2). Studies have shown that TTM improves neurological outcomes in patients after cardiac arrest (3).

The neuroprotective effects of TTM can be influenced by several factors, including the initial rhythm of cardiac arrest, pre-admission ROSC, the provision of percutaneous coronary intervention, the cooling method for the maintenance phase of TTM, and bystander cardiopulmonary resuscitation (BCPR) (4, 5). BCPR provides blood circulation to vital organs after cardiac arrest, thus reducing the risk of brain damage. The survival and neurological benefits of BCPR have been rigorously investigated in existing literature (6). However, the outcome benefits of BCPR in patients receiving TTM have not been explored. This study, therefore, aimed to investigate the outcome differences between BCPR and no-BCPR (those who had not received BCPR) in patients receiving TTM after cardiac arrest.

## Materials and Methods

### Study Design and Setting

We conducted a retrospective observational cohort study from January 2013 to September 2019 using data from the Taiwan Network of Targeted Temperature Management for Cardiac Arrest (TIMECARD) registry, a nationwide multicenter registry for cardiac arrest patients receiving TTM in post-cardiac arrest care (7).

The TIMECARD registry was managed by the Taiwan Society of Emergency and Critical Care Medicine. In participating hospitals, TTM was provided to patients with out-of-hospital cardiac arrest (OHCA) and in-hospital cardiac arrest (IHCA) with a Glasgow Coma Scale (GCS) score of <8 or those who could not obey verbal commands after ROSC. A temperature range between 32 and 36°C was maintained for at least 24 h, after which the body was slowly rewarmed at a rate of 0.2–0.5°C/h. Patients aged ≥ 18 years who received TTM after ROSC were included in this study.

### Data Collection and Definitions

The primary variable in this study was whether the patient had received BCPR. In this study, BCPR was defined as “an attempt to perform basic cardiopulmonary resuscitation by someone who is not a part of an organized emergency response system,” according to the Utstein templates for resuscitation (8). Covariates of patient-related factors such as age, sex, and comorbidities were included. Covariates of resuscitation parameters such as event time, event location, witnessed collapse, initial rhythms, cause of cardiac arrest, cardiopulmonary resuscitation (CPR) duration, electrical discharge therapy, and pre-hospital ROSC were also included.

The initial rhythm was determined using either a manual defibrillator or an automated external defibrillator (AED). Cardiac arrest is classified as cardiogenic or non-cardiogenic. Cardiogenic origin is defined as cardiac arrest caused by myocardial ischemia or infarction, hypertrophic cardiomyopathy, valvular disease, and heart failure. Non-cardiac causes include drowning, trauma, asphyxia, respiratory disease, malignancy, electrolyte imbalance, sepsis, and uncontrolled bleeding. Electrical discharge therapy included defibrillation and synchronized cardioversion with manual defibrillators or AEDs. ROSC was defined as a palpable pulse lasting >20 s. Blood pressure and heart rate were immediately measured after ROSC.

We also recorded the heart rate, mean arterial pressure, and the GCS score at ROSC, time from ROSC to targeted temperature range, the cooling method for the maintenance phase of TTM, cold saline infusion during TTM, and patients who received coronary angiography. The cooling methods for the maintenance phase of TTM were classified into external and internal cooling. External cooling included a traditional cold blanket and the Arctic Sun medical device, which modulates patient temperature by circulating cold water in pads directly adhered to the patient's skin. Internal cooling included an intravascular cooling device and extracorporeal membrane oxygenation (ECMO).

### Outcomes Measures and Statistical Analysis

Results are expressed as n (%) for categorical variables. Descriptive statistics were reported as mean and standard deviation for continuous variables. The groups were compared using Pearson's chi-squared test for categorical data and Student's *t-*test for numerical data. We compared the survival, the GCS score, and the Glasgow–Pittsburgh cerebral performance category (CPC) scale score between the BCPR group and the no-BCPR group while transferring out of ICU and again during hospital discharge. A comparison of mortality rates between BCPR and no-BCPR patients was also analyzed in a different subgroup. The multivariate logistic regression model was used to explore independent risk factors for in-hospital mortality. Odds ratio (OR) and 95% confidence interval (CI) were identified for each risk factor. Important significant risk factors were identified using the stepwise logistic regression model. All data were processed using SAS software (version 9.4; SAS Institute Inc., Cary, NC). A *P* < 0.05 was considered statistically significant.

## Results

### Study Population

A total of 580 patients were enrolled in this study, of which 376 were in the BCPR group, and the remaining 204 were in the no-BCPR group. The basic characteristics of cardiac arrest patients who received TTM in the BCPR and no-BCPR groups are listed in Table 1. The mean age was 62.1 in the BCPR group and 67.3 in the no-BCPR group (Table 1).

**Table 1:** Basic characteristics for cardiac arrest patients receiving TTM between BCPR group and NO-BCPR group.

**Variables**	**BCPR group (*****N*** **=** **376)**		**NO-BCPR group (*****N*** **=** **204)**		***P*-Value**
Male	251	(66.76%)	130	(63.73%)	0.4630
Age ≤ 65 years	182	(48.40%)	98	(48.04%)	0.9330
Comorbidities
Diabetes mellitus	162	(43.09%)	77	(37.75%)	0.2122
Hypertension	218	(57.98%)	109	(53.43%)	0.2917
Coronary artery disease	104	(27.66%)	50	(24.51%)	0.4121
Dyslipidemia	74	(19.68%)	33	(16.18%)	0.2988
Heart failure	70	(18.62%)	40	(19.61%)	0.7713
Arrhythmia	52	(13.83%)	19	(9.31%)	0.1131
Chronic kidney disease	72	(19.15%)	33	(16.18%)	0.3747
ESRD under dialysis	47	(12.50%)	23	(11.27%)	0.6653
Malignancy	56	(14.89%)	17	(8.33%)	0.0229
Event time
Workday	241	(64.10%)	119	(58.33%)	0.1720
Weekend	135	(35.90%)	85	(41.67%)	
Event location
Out-of-hospital cardiac arrest	281	(74.73%)	188	(92.16%)	**<0.0001**
In-hospital cardiac arrest	95	(25.27%)	16	(7.84%)	
Witnessed collapse	341	(90.69%)	125	(61.27%)	**<0.0001**
Initial rhythm
Shockable	142	(37.77%)	68	(33.33%)	0.2888
Non-shockable	234	(62.23%)	136	(66.67%)	
Cause of cardiac arrest
Cardiac	207	(55.05%)	98	(48.04%)	0.1062
Non-cardiogenic	169	(44.95%)	106	(51.96%)	
CPR duration > 10 min	285	(75.80%)	166	(81.37%)	0.1232
Electrical discharge therapy	153	(40.69%)	79	(38.73%)	0.6444
Pre-Hospital ROSC	69	(18.35%)	48	(23.53%)	0.1378
Heart rate (bpm) at ROSC
<100	173	(46.01%)	90	(44.12%)	0.6619
≥100	203	(53.99%)	114	(55.88%)	
MAP (mmHg) at ROSC
<65	66	(17.55%)	36	(17.65%)	0.9774
≥65	310	(82.45%)	168	(82.35%)	
Glasgow coma scale (GCS) at ROSC
<8	368	(97.87%)	199	(97.55%)	0.7766
≥8	9	(2.39%)	5	(2.45%)	
Time from ROSC to targeted temperature
<12 h	252	(69.23%)	127	(65.13%)	0.3225
≥12 h	112	(30.77%)	68	(34.87%)	
Method for maintenance phase of TTM
External cooling	332	(88.30%)	190	(93.14%)	0.0636
Internal cooling	44	(11.70%)	14	(6.86%)	
Cold saline infusion during TTM	148	(39.36%)	98	(48.04%)	0.0435
Received coronary angiography	127	(33.78%)	58	(28.43%)	0.1872

*Values are expressed as numbers (percentage)*.

*BCPR, bystander cardiopulmonary resuscitation; bpm, beats per minute; CPR, cardiopulmonary resuscitation; ESRD, end stage renal disease; MAP, mean arterial pressure; ROSC, return of spontaneous circulation; TTM, targeted temperature management*.

### Effect of BCPR on Survival and Neurological Outcomes in ROSC Patients Post-TTM

A survival benefit was found in the BCPR group. Compared to those in the no-BCPR group, BCPR patients who received TTM after ROSC had a higher survival rate at hospital discharge (42.25 vs. 31.86%, *P* = 0.0305, Table 2). However, there was no significant difference in the survival rate of patients in the BCPR and no-BCPR groups (52.66 vs. 49.51%, *P* = 0.4686, Table 2) while transferring out of ICU.

**Table 2:** Survival and neurological outcomes for cardiac arrest patients receiving TTM between BCPR group and NO-BCPR group.

***Outcomes***	**BCPR group (*****N*** **=** **376)**		**NO-BCPR group (*****N*** **=** **204)**		***P*-Value**
Survived at transferring out of ICU	198	(52.66%)	101	(49.51%)	0.4686
GCS at transferring out of ICU
GCS <8	87	(44.16%)	65	(64.36%)	**0.0010**
GCS ≥ 8	110	(55.84%)	36	(35.64%)	
Average (SD)	9.83	(4.77)	6.76	(4.05)	**<0.0001**
CPC at transferring out of ICU
CPC 1–2	90	(45.69%)	25	(24.75%)	**0.0004**
CPC 3–5	107	(54.31%)	76	(75.25%)	
Average (SD)	2.51	(1.26)	3.28	(1.04)	**<0.0001**
Survived at hospital discharge	158	(42.25%)	65	(31.86%)	**0.0305**
GCS at hospital discharge
GCS <8	57	(34.55%)	30	(44.12%)	0.1697
GCS ≥ 8	108	(65.45%)	38	(55.88%)	
Average (SD)	11.3	(4.58)	8.31	(4.37)	**<0.0001**
CPC at hospital discharge
CPC 1–2	91	(55.15%)	26	(38.24%)	**0.0189**
CPC 3–5	74	(44.85%)	42	(61.76%)	
Average (SD)	2.14	(1.27)	2.98	(1.18)	**<0.0001**

*Values are expressed as mean (standard deviation) for continuous variables and numbers (percentage) for categorical variables*.

*BCPR, bystander cardiopulmonary resuscitation; CPC, Cerebral Performance Categories; GCS, Glasgow Coma Scale; ICU, intensive care unit; SD, standard deviation; TTM, targeted temperature management. Statistically significant data (P < 0.05) were expressed as bold values*.

The BCPR group also had a better neurological outcome while transferring out of ICU. The BCPR group had more patients with GCS ≥8 than the no-BCPR group (55.84 vs. 35.64%, *P* = 0.0010, Table 2). Their average GCS score was higher (9.83 vs. 6.76, *P* < 0.0001, Table 2), and more patients in BCPR group were scored 1–2 on the CPC scale (45.69 vs. 24.75%, *P* = 0.0004, Table 2). The average CPC scale was also lower in the BCPR group (2.51 vs. 3.28, *P* < 0.0001, Table 2).

The data collected at hospital discharge also showed neurological benefits in patients in the BCPR group. The group had a higher average GCS score (11.3 vs. 8.31, *P* < 0.0001, Table 2) and a lower average CPC scale score (2.14 vs. 2.98, *P* < 0.0001, Table 2).

### Effect of BCPR on Survival in Different Subgroups in ROSC Patients Post-TTM

The effect of BCPR on post-TTM survival in various subgroups is presented in Table 3. Compared to the patients in the no-BCPR group, the BCPR group had a lower mortality rate in male patients (50.60 vs. 65.38%, *P* = 0.0059), patients aged >65 (59.28 vs. 74.53%, *P* = 0.0083), patients with cardiac arrest on workday (53.11 vs. 64.71%, *P* = 0.0366), and patients with OHCA (54.09 vs. 64.36%, *P* = 0.0271). While studying BCPR patients with co-morbidities, a survival benefit was also found among patients with hypertension (56.42 vs. 67.89%, *P* = 0.0458), patients with dyslipidemia (55.41 vs. 78.79%, *P* = 0.0210), patients without chronic kidney disease (CKD) (51.64 vs. 61.40%, *P* = 0.0401), patients without end-stage renal disease (ESRD) under dialysis (51.37 vs. 61.88%, *P* = 0.0224), and patients without malignancy (53.13 vs. 63.64%, *P* = 0.0211). Two resuscitation-related parameters, heart rate <100 bpm at ROSC (52.02 vs. 66.67%, *P* = 0.0228) and mean arterial pressure (MAP) ≥65 mmHg at ROSC (50.65 vs. 61.90%, *P* = 0.0182), were associated with a lower mortality rate in the BCPR group.

**Table 3:** In-hospital mortality rates in different subgroups between BCPR group and NO-BCPR group.

**Subgroups**		**BCPR (*****N*** **=** **376)**		**NO-BCPR (*****N*** **=** **204)**		**Subgroup *P*-value**
		**Death/Total**	**Mortality**	**Death/Total**	**Mortality**	
Sex	Male	127/251	(50.60%)	85/130	(65.38%)	**0.0059**
	Female	80/125	(64.00%)	45/74	(60.81%)	0.6528
Age	≤65	92/182	(50.55%)	51/98	(52.04%)	0.8118
	>65	115/194	(59.28%)	79/106	(74.53%)	**0.0083**
Event time	Weekend	79/135	(58.52%)	53/85	(62.35%)	0.5719
	Workday	128/241	(53.11%)	77/119	(64.71%)	**0.0366**
Event location	OHCA	152/281	(54.09%)	121/188	(64.36%)	**0.0271**
	IHCA	55/95	(57.89%)	9/16	(56.25%)	0.9020
Diabetes mellitus	NO	107/214	(50.00%)	73/127	(57.48%)	0.1810
	YES	100/162	(61.73%)	57/77	(74.03%)	0.0613
Hypertension	NO	84/158	(53.16%)	56/95	(58.95%)	0.3703
	YES	123/218	(56.42%)	74/109	(67.89%)	**0.0458**
Coronary artery disease	NO	148/272	(54.41%)	96/154	(62.34%)	0.1121
	YES	59/104	(56.73%)	34/50	(68.00%)	0.1806
Dyslipidemia	NO	166/302	(54.97%)	104/171	(60.82%)	0.2167
	YES	41/74	(55.41%)	26/33	(78.79%)	**0.0210**
Heart failure	NO	161/306	(52.61%)	100/164	(60.98%)	0.0821
	YES	46/70	(65.71%)	30/40	(75.00%)	0.3107
Arrhythmia	NO	179/324	(55.25%)	115/185	(62.16%)	0.1287
	YES	28/52	(53.85%)	15/19	(78.95%)	0.0554
Chronic kidney disease	NO	157/304	(51.64%)	105/171	(61.40%)	**0.0401**
	YES	50/72	(69.44%)	25/33	(75.76%)	0.5062
ESRD under dialysis	NO	169/329	(51.37%)	112/181	(61.88%)	**0.0224**
	YES	38/47	(80.85%)	18/23	(78.26%)	1.0000
Malignancy	NO	170/320	(53.13%)	119/187	(63.64%)	**0.0211**
	YES	37/56	(66.07%)	11/17	(64.71%)	0.9172
Witnessed collapsed	NO	25/35	(71.43%)	56/79	(70.89%)	0.9530
	YES	182/341	(53.37%)	74/125	(59.20%)	0.2626
Initial rhythm	Non-shockable	147/234	(62.82%)	97/136	(71.32%)	0.0961
	Shockable	60/142	(42.25%)	33/68	(48.53%)	0.3916
Electrical discharge	NO	141/233	(60.52%)	89/125	(71.20%)	0.1318
	YES	66/153	(43.14%)	41/79	(51.90%)	0.2046
Prehospital ROSC	NO	181/307	(58.96%)	106/156	(67.95%)	0.0596
	YES	26/69	(37.68%)	24/48	(50.00%)	0.1852
Heart rate at ROSC	<100 bpm	90/173	(52.02%)	60/90	(66.67%)	**0.0228**
	≥100 bpm	117/203	(57.64%)	70/114	(61.40%)	0.5127
MAP at ROSC	<65 mmHg	50/66	(75.76%)	26/36	(72.22%)	0.6954
	≥65 mmHg	157/310	(50.65%)	104/168	(61.90%)	**0.0182**
Cause of cardiac arrest	Non-cardiogenic	108/169	(63.91%)	74/106	(69.81%)	0.3137
	Cardiogenic	99/207	(47.83%)	56/98	(57.14%)	0.1285

*Values are expressed as numbers (percentage)*.

*BCPR, bystander cardiopulmonary resuscitation; bpm, beats per minute; ESRD, end stage renal disease; MAP, mean arterial pressure; ROSC, return of spontaneous circulation. Statistically significant data (P < 0.05) were expressed as bold values*.

### Independent Risk Factors of In-Hospital Mortality in Patients With Cardiac Arrest Receiving TTM

We performed a multivariate logistic regression analysis to explore independent risk factors for in-hospital mortality. The adjusted odds ratio and 95% confidence interval of each risk factor are shown in Table 4. Bystander CPR was a significant positive predictor, with an adjusted odds ratio (OR) of 0.66 (95% CI: 0.45–0.97, *P* = 0.0363, Table 4) for in-hospital mortality. On the other hand, the unadjusted odds ratio of bystander CPR was 0.697 (95% CI 0.49–0.99, *P* = 0.0436).

**Table 4:** Independent risk factors of in-hospital mortality in cardiac arrest patients receiving TTM.

**Variables**	**OR**	**(95% CI)**	***P*-value**
**Multivariate logistic regression**
Male sex	1.07	(0.72–1.60)	0.7457
Age > 65	1.36	(0.92–2.01)	0.1265
Event time: weekend	1.19	(0.82–1.74)	0.3576
Diabetes mellitus	1.32	(0.87–2.02)	0.1976
Hypertension	0.89	(0.59–1.34)	0.5740
Coronary artery disease	1.24	(0.79–1.94)	0.3591
Heart failure	1.31	(0.78–2.22)	0.3078
Arrhythmia	0.82	(0.45–1.48)	0.5055
Chronic kidney disease	1.30	(0.76–2.22)	0.3384
ESRD under dialysis	2.53	(1.30–4.90)	**0.0061**
Dyslipidemia	1.12	(0.66–1.91)	0.6661
Malignancy	1.37	(0.78–2.40)	0.2772
Bystander CPR	0.66	(0.45–0.97)	**0.0363**
AED defibrillation	1.46	(0.66–3.21)	0.3531
Initial shockable rhythm	0.63	(0.27–1.47)	0.2822
Electrical discharge	0.86	(0.36–2.06)	0.7309
Prehospital ROSC	0.55	(0.35–0.88)	**0.0123**
Heart rate at ROSC ≥100 bpm	1.11	(0.76–1.62)	0.5866
MAP at ROSC <65 mmHg	2.54	(1.52–4.25)	**0.0004**
Cardiogenic cardiac arrest	0.99	(0.61–1.62)	0.9696
Cold saline infusion	0.79	(0.54–1.15)	0.2138
Coronary angiography	0.48	(0.29–0.81)	**0.0056**
**Stepwise multiple regression**
ESRD under dialysis	2.96	(1.57–5.58)	**0.0008**
Bystander CPR	0.67	(0.46–0.98)	**0.0391**
Prehospital ROSC	0.50	(0.32–0.77)	**0.0020**
MAP at ROSC <65 mmHg	0.42	(0.25–0.69)	**0.0006**
Coronary angiography	0.37	(0.25–0.54)	**<0.0001**

*AED, automated external defibrillator; CPR, cardiopulmonary resuscitation; bpm, beats per minute; ESRD, end stage renal disease; MAP, mean arterial pressure; ROSC, return of spontaneous circulation. Statistically significant data (P < 0.05) were expressed as bold values*.

In addition to bystander CPR, prehospital ROSC (OR = 0.55, 95% CI: 0.35–0.88, *P* = 0.0123, Table 4) and coronary angiography (OR = 0.48, 95% CI: 0.29–0.81, *P* = 0.0056, Table 4) were significant positive predictors in multivariate logistic regression model. On the contrary, ESRD under dialysis (OR = 2.53, 95% CI: 1.30–4.90, *P* = 0.0061, Table 4) and mean arterial pressure at ROSC <65 mmHg (OR = 2.54, 95% CI: 1.52–4.25, *P* = 0.00004, Table 4) were significant negative predictors. Stepwise logistic regression for important factors was also analyzed. The odds ratio for each important factor is demonstrated in Table 4.

## Discussion

### BCPR Improved Survival in Patients Post-TTM

This study aimed to investigate the outcome differences between BCPR and no-BCPR patients receiving TTM after cardiac arrest. Among patients who received TTM, BCPR was associated with a higher survival rate until hospital discharge than those who did not receive BCPR (42.25 vs. 31.86%, *P* = 0.0305, Table 2). The positive effects of BCPR on OHCA patients have been extensively investigated. According to a meta-analysis of 16 cohort studies, BCPR was associated with an ~2-fold chance of survival of patients with OHCA compared to patients who received no-BCPR (OR = 1.95; 95% CI: 1.66–2.30) (6). Notably, our study proved the survival benefit of BCPR in patients with cardiac arrest, specifically in those receiving TTM. During cardiac arrest, the cerebral blood flow is extremely low (9). If patients with cardiac arrest received BCPR, they could have better cerebral blood flow, which consequently contributes to improved survival outcomes after TTM.

The positive survival impact of BCPR was significant among older patients (aged > 65 years) undergoing TTM (59.28 vs. 74.53%, *P* = 0.0083, Table 3). In a previous study, BCPR had a higher OR for 1-month survival in patients aged >71 years (OR = 5.1, 95% CI: 3.8–7.1) than in those aged ≤71 years (OR = 2.5, 95% CI: 1.9–3.3) (10). In terms of their response to TTM, a previous study showed that TTM was significantly associated with good neurologic outcomes in patients aged <65 years but had no association with outcomes in older patients (65–74 years: OR 1.49, 95% CI: 0.90–2.47; >75 years: OR 1.44, 95% CI: 0.79–2.34) (11). Although older patients may not benefit much from TTM, they can have better survival outcomes after receiving BCPR.

Our study showed that patients who received BCPR had a better chance of survival in post-TTM care when they did not have CKD (51.64 vs. 61.40%, *P* = 0.0401, Table 3), ESRD on dialysis (51.37 vs. 61.88%, *P* = 0.0224, Table 3), and malignancy (53.13 vs. 63.64%, *P* = 0.0211, Table 3). A previous study also suggested that BCPR had a stronger survival impact on patients with less severe comorbidities (12). One possible explanation is that additional comorbidities may hasten the electrical, hemodynamic, and metabolic decline in patients with cardiac arrest, making BCPR less effective in rescuing a patient (13).

Our findings showed that BCPR improved survival in patients with OHCA (54.09 vs. 64.36%, *P* = 0.0271, Table 3) but did not show a survival benefit in patients with IHCA. In patients who did not receive BCPR, the interval time between OHCA and EMS arrival was longer than the interval time between IHCA and CPR provided by the healthcare team. BCPR could significantly reduce the time from arrest to first CPR in patients with OHCA but not in patients with IHCA. This could be a possible reason why BCPR has different effects on survival between patients with OHCA and IHCA.

### BCPR Preserved Pre-arrest Neurological Status in Patients Post-TTM

The result of our study demonstrated that BCPR was associated with better neurological outcomes. Similar results were reported in a previous study. According to a meta-analysis in 2018, favorable neurological outcomes were associated with a significantly higher odds ratio of BCPR (OR, 1.44; 95% CI: 1.14–1.82) in patients treated with TTM after cardiac arrest (14). Given that immediate CPR provides crucial blood flow to the brain and shortens ischemia time, BCPR has a positive impact on neurological outcomes in cardiac arrest patients treated with TTM. Therefore, community interventions to encourage BCPR should be undertaken to improve the functional outcomes of patients with cardiac arrest.

### Other Independent Risk Factors of In-Hospital Mortality in Patients Post-TTM

Preexisting comorbidities in patients with cardiac arrest influenced their survival after TTM therapy. When adjusted for other variables, ESRD under dialysis (OR = 2.53, 95% CI: 1.30–4.90, Table 4) was an independent negative predictive factor for survival. Hirlekar et al. also demonstrated that renal disease (OR = 0.53, 95% CI: 0.53–0.72) reduced the chance of 30-d survival of patients with OHCA (15).

Pre-hospital ROSC (OR = 0.55, 95% CI: 0.35–0.88, Table 4) was also an independent prognostic factor for survival in our study. Since chest compressions only generate 25–30% of the normal cardiac output (16), prolonged CPR increases cerebral damage (17). Therefore, a pre-hospital ROSC could result in good survival among TTM recipients by reducing the levels of brain damage prior to TTM.

Coronary angiography (OR = 0.48, 95% CI: 0.29–0.81, Table 4) had a positive effect on in-hospital survival in BCPR patients. Acute coronary syndrome is a major cause of OHCAs, requiring emergency coronary angiography for immediate diagnosis and treatment. Although immediate coronary angiography could delay TTM therapy for ~1 h (18), current guidelines recommend immediate coronary angiography and percutaneous coronary intervention in resuscitated OHCA patients whose ECGs show ST-elevation myocardial infarction (19). Given that hemodynamic instability and cardiac dysfunction could worsen during TTM, percutaneous coronary intervention could provide better outcomes in TTM recipients by allowing revascularization of the coronary artery and supporting the hemodynamic status during post-resuscitation care (4).

Mean arterial pressure at ROSC <65 mmHg was an independent negative predictor of in-hospital mortality in patients post-TTM. A previous retrospective cohort study also found that post-ROSC hypotension was an independent predictor of survival among patients who had ROSC after OHCA (20).

### Study Limitations

Our study is subject to certain limitations. First, this was a retrospective, non-randomized study. Potential selection bias may exist due to differences in basic patient characteristics between the control and experimental groups. However, selection bias was limited by the large sample size in this study. Second, our analyses were based on observational data. Therefore, although our study showed correlations between BCPR, survival, and neurological outcomes, we could not prove causality. Third, TTM duration, targeted temperature, and cooling methods differed between hospitals due to their differing protocols. This may be an unknown bias that influences the overall survival and neurological outcomes.

### Study Strengths

This is the first nationwide multicenter registry project to compare survival and neurological outcomes between cardiac patients under TTM care who had received BCPR with those who did not receive BCPR. A significant positive survival impact of BCPR was found in multiple subsets in the subgroup analysis. This study also identified independent risk and protective factors and their odds ratio for in-hospital mortality among patients who received TTM. Since this study proved that BCPR increases the neuroprotective effects of TTM, the public should be encouraged to offer more BCPR, which will improve post-TTM outcomes in cardiac patients.

## Conclusions

This study demonstrated that BCPR had a positive survival and neurological outcome on the return of spontaneous circulation in patients post-TTM.

## Data Availability Statement

The original contributions presented in the study are included in the article/supplementary material, further inquiries can be directed to the corresponding author/s.

## Ethics Statement

The studies involving human participants were reviewed and approved by the Institutional Review Board (IRB) of the Kaohsiung Veterans General Hospital approved this study (No. VGHKS18-EM3-02). Written informed consent for participation was not required for this study in accordance with the national legislation and the institutional requirements.

## Author Contributions

W-CH: conception, study design, and critical revision of the manuscript. M-ST, L-KK, H-HH, C-HL, and K-CL: data acquisition, analysis, and interpretation, F-YL: drafting of the manuscript. All authors have read and approved the final manuscript.

## Funding

This study was supported by grants from the Kaohsiung Veterans General Hospital, Kaohsiung, Taiwan (VGHKS19-EM12-01).

## Conflict of Interest

The authors declare that the research was conducted in the absence of any commercial or financial relationships that could be construed as a potential conflict of interest.

## Publisher's Note

All claims expressed in this article are solely those of the authors and do not necessarily represent those of their affiliated organizations, or those of the publisher, the editors and the reviewers. Any product that may be evaluated in this article, or claim that may be made by its manufacturer, is not guaranteed or endorsed by the publisher.
